# Epidemiology of Depression and Associated Factors among Asthma Patients in Addis Ababa, Ethiopia

**DOI:** 10.1155/2018/5934872

**Published:** 2018-08-26

**Authors:** Mebrat Abera Woledesenbet, Shegaye Shumet Mekonen, Lamesa Melese Sori, Tadesse Melaku Abegaz

**Affiliations:** ^1^University of Gondar Hospital, College of Medicine and Health Sciences, Department of Psychiatry, Ethiopia; ^2^University of Gondar, College of Medicine and Health Sciences, Department of Psychiatry, Ethiopia; ^3^University of Gondar, College of Medicine and Health Sciences, Department of Clinical Pharmacy, Ethiopia

## Abstract

**Background:**

Depression in asthma patients can cause worsening of respiratory symptoms. Addressing mental illness in those with asthma improves asthma outcomes. This study aimed to assess the epidemiology of depression and associated factors among asthma patients attending government hospitals in Ethiopia.

**Methods:**

Institutional based cross-sectional study was conducted on patients with asthma at three governmental hospitals of Addis Ababa from June to July 2017. Patient health questionnaire (PHQ-9) depression scale was used to assess prevalence of depression among asthmatic patients. The data were entered and analyzed using SPSS version 20 statistical software. Binary logistic regression analysis was conducted to identify associated factors for depression. To indicate the strength of association, odds ratios (OR) and 95% confidence intervals (95% CI) were used.

**Result:**

A total of 405 participants were enrolled in the study giving an overall response rate of 96%. The respondents had mean age of 54.46 and standard deviation (SD) of 10.01 years. About 273 (67.4%) were females. The prevalence of depression among asthma patients was 85 (21%). The odds of developing depression among single asthma patients were increased by 1.63 with 95% CI [1. 8, 3.493]. Depression among asthma patients who had comorbid cardiac illness was 6.2 times higher than those who do not have at CI [1.145, 24.109]. The prevalence of depression among uncontrolled asthma patients was 8 times higher than those with well-controlled asthma at CI [1.114, 19.025].

**Conclusion:**

One-fifth of asthmatic patients were experiencing depression. Uncontrolled asthma, comorbid cardiac illness, and single patients were important predictors of depression among asthmatic patients. Proper control of asthmatic attack and cardiac illnesses is very important to reduce the burden of depression.

## 1. Introduction 

Depression is a common mental disorder that presents with depressed mood, loss of interest or pleasure, decreased energy, feelings of guilt or low self-worth, disturbed sleep or appetite, and poor concentration [1–3]. Depression is a significant contributor to the global burden of disease and affects people in all communities across the world. Today, depression is estimated to affect 350 million people [3]. Depression is predicted to be the second leading cause of global disability burden by 2020 [4]. Depressive disorders are at least twice as common in patients with asthma when compared to the general population [5]. Depression appears frequently in cases of bronchial asthma and much higher than that of the healthy population. Depression is significantly higher with uncontrolled asthma than controlled [6]. Patients with more severe asthma were more likely to be at risk for depression [7]. Depression in asthma patients can cause a worsening of respiratory symptoms and an increase of the disease exacerbations [8, 9]. Depression is also a risk factor for developing severe life-threatening asthma [10].

According to world health survey in 57 countries, depression among asthma patients in Europe was 7.3%, Australia 6.6%, South America 17.1, Asia 7.1%, and Africa 8% [11]. In the year 2013, about 17% of elderly patients with asthma encountered clinically significant symptoms of depression in United States of America (USA) [12]. A cross-sectional study done in 2011 on French asthma patients found a 9.6% of rate of depression [13]. In Nigeria, the prevalence of depression was 67.4% [14]. The prevalence of depression was found to range from 15% to 46% among asthma patients in Egypt [15]. The pooled prevalence of depression in Ethiopia was reported to be 6.8% (95%, CI: 6.4-7.3) [16].

Epidemiology of depression among asthmatic patients was different based on independent predicting factors. Pronounced sex differences were found in South America and in Asia with higher odds ratio in men than in women [11]. Depression was more likely associated with female gender in USA patients [12] but the duration of asthma diagnosis or intubation rates did not affect magnitude of depression in these patients. The 2012 world health survey did not identify a considerable difference of depression among gender [17]. Poor control of asthma showed increased risk of depression [4]. Further, unemployment and lower economic status were highly associated with depression in Korean asthma patients [18]. A study done in Iran revealed that depression was more associated with severe asthma [18]. In Ethiopia, sex, age, marital status, violence, migration, and substance use were associated with depression [16].

Coincidence of depression with asthma affects the quality of life, and disease controls social and financial aspects of individuals. Depression creates a substantial personal burden for affected individuals and their families, including significant economic and social hardships. Early detection and treatment of depressive disorders are vital to reduce morbidity and mortality. Therefore recognizing prevalence of depression and associated factors among asthmatic patients is important for designing early and appropriate intervention. Data were scarce on the prevalence of depression among asthmatic patients on this study area. Therefore, this study sought to assess the prevalence of depression and associated factors among asthma patients attending government hospitals located in Addis Ababa, Ethiopia.

## 2. Methods and Materials

### 2.1. Study Area and Study Period

The study was conducted in Addis Ababa governmental hospitals from June to July 2017; Addis Ababa is the capital city of Ethiopia, and there are 5 federal, 6 regional, and 2 army hospitals. Asthma patients from 3 government hospitals, namely, Tikur Anbessa Referral Hospital, St. Paul's Millennium Medical College Referral Hospital, and Yekatit 12 Medical College Referral Hospital, were included. Tikur Anbessa Hospital had an average 168 asthma patients, Saint Paul's Millennium Medical College Referral Hospital had an average 149 asthma patients, and Yekatit 12 Medical College Referral Hospital had an average 105 asthma patients, who have regular monthly follow-up.

### 2.2. Study Design and Population

Institution based cross-sectional study design was conducted on asthma patients who were attending Addis Ababa governmental hospitals and have follow-up. The study population was asthma patients who have follow-up visit during the data collection period. Age was limited to above 18 years during the data collection period. Patients who had a series medical illness that needs an emergency care and were unable to communicate were excluded.

### 2.3. Sample Size Determination and Sampling Procedure

All asthma patients who had a regular attendance at aforementioned hospitals were included. Therefore, all asthmatic patients attending the following hospitals Tikur Anbessa 168, Saint Paul 149, and Yekatit 105 were included. Participants of this study were selected using a convenient sampling technique. The three hospitals were randomly selected using lottery method.

#### 2.3.1. Study Variables

Presence of depression based on the objective criteria was our dependent variables. The independent variables included age, sex, religion, ethnicity, marital status, occupational status, educational status, past psychiatric illness, family psychiatry, history, type of medication, level of asthma symptom control, and substance use.

#### 2.3.2. Operational Definitions

Depression based on PHQ-9 depression scale among asthmatic patients who score ≥10 was considered as depressive [17]. Current substance use refers to the use of alcohol, khat, and cigarette for the past three months. Chronic medical illness means proven medical illness (hypertension, diabetes mellitus, and cardiac illness). Social support, based on Oslo social support scale, with score 3-8 was considered as poor support, 9-11 moderate support, and 12-14 strong support [19]. Level of asthma symptom control based on global initiative for asthma (GINA) guideline is as follows: symptom within 4 weeks, none of the symptoms considered as controlled, 1-2 symptoms considered as partly controlled, and 3-4 symptoms considered as uncontrolled [20].

#### 2.3.3. Data Collection Method and Data Collection Procedures

Quantitative data was collected by semistructured interview questionnaire and has five parts. The 1^st^ part contains sociodemographic characteristics of participants; the 2^nd^ part contains PHQ 9 depression scale; the 3^rd^ part contains substance related factors; the 4^th^ part contains clinical factors; the 5^th^ part was psychosocial factors. All parts were translated to Amharic language.

#### 2.3.4. Data Quality Control

To assure the quality of data, high emphasis was given in designing data collection instruments for its simplicity and pretest was done two weeks before the actual data collection and some modification was made accordingly. Training on data collection instrument was given to data collectors and supervisors by the principal investigator. The collected data was reviewed and checked for completeness and relevance by supervisor and principal investigator each day.

#### 2.3.5. Data Processing and Analysis

After the data was collected, it was coded, edited, cleaned, and entered in to Epi data version 3.1 and analyzed using SPSS version 20. Bivariate analyses were also used to explore relationship between the outcome variable and the independent variables with a value of p≤0.25 for multivariate logistic regression for further analysis. Furthermore, to control the effects of confounding variables a multivariate logistic regression was fit. Odds ratio and 95% confidence intervals were set. For the purpose of this study, p value less than 0.05 was taken as statistical significant.

## 3. Result

A total of 405 participants were enrolled in the study giving an overall response rate of 96%. The respondents had mean age of 54.46 (SD=10.01) years and 273 (67.4%) were females and 132 (32.6%) were male. Regarding the religion majority 285 (70.4%) of the respondents were orthodox and 84 (20.7%) were Muslim. About 180 (44.4%) were Oromo and 134 (33.2%) were Amhara. Most of the study subjects 153 (37.8%) were unable to read and write. About 191 (47.2%) were housewives and 70 (17.3%) were retired. Majority 282 (69.6%) were married and 82 (20.2%) were widowed (Table 1).

### 3.1. Clinical, Psychosocial, and Substance Related Factors

About 401 (99%) of the respondents were not having past psychiatric history and 20 (4.9%) of the respondents had family psychiatric history. About 78 (19.3%) of the respondents were found to have another medical condition. Majority 42 (53.8%) have hypertension followed by 20 (25.6%) having cardiac illness and 16 (16.7%) having diabetes. All respondents were on medications for asthma. About 331 (81.7%) were on salbutamol, 169 (41.7%) were on beclomethasone, 135 (33.3%) were on both salbutamol and beclomethasone, the remaining 50 (12.3%) were on prednisolone, and 13 (3.2%) were on other asthma medications. Regarding level of asthma symptom control, majority 170 (42%) were partially controlled, 126 (31.1%) were uncontrolled, and 109 (26.9%) were found to have well-controlled asthma. About 24 (5.9%) of the respondents had history of use substance, 20 (4.9%) of them used alcohol, and 5 (1.2%) used other (khat, cigarette). About 211 (52.1%) had poor social support, 138 (34.1%) had moderate social support, and 56 (13.8%) had strong social support. Around 15 (3.7%) had history of close family death (Table 2).

### 3.2. Prevalence of Depression

**Figure 1:**
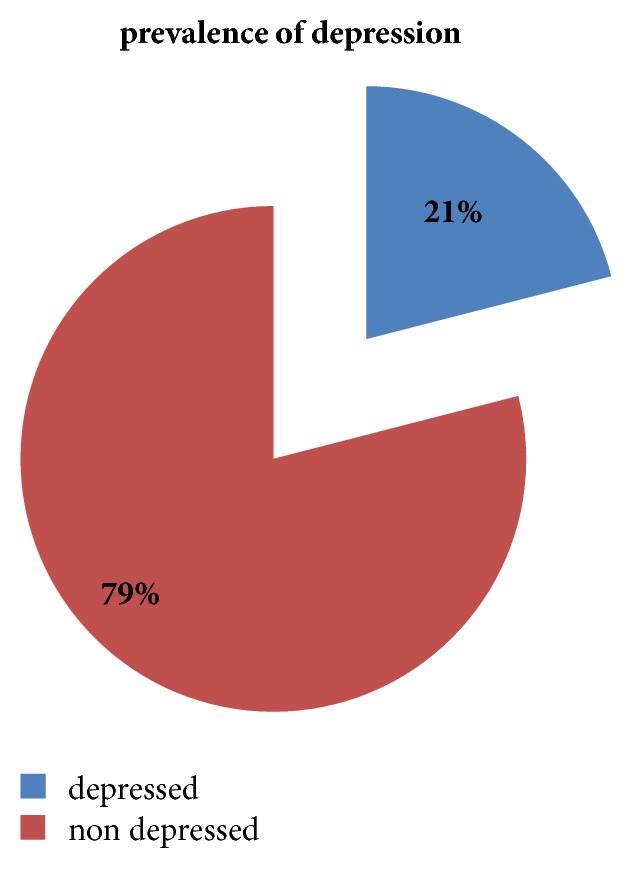
Prevalence of depression among asthma patients at governmental hospitals Addis Ababa, Ethiopia, 2017.

The overall prevalence of depression among patients with asthma was found to be 85 (21%) (Figure 1).

### 3.3. Factors Associated with Depression among Asthmatic Patients

After bivariate logistic regression analysis, each independent variable to dependent variable with p values less than or equal to 0.25 was entered into multivariate logistic regression analysis for further analysis between independent and dependent variables and p value less than 0.05 was taken as significant. From bivariate analysis, sex, marital status, substance use, past psychiatric history, type of asthma medication (beclomethasone, prednisolone), cardiac illness, and asthma symptom control level were factors associated with depression among asthmatic patients and entered in multivariate logistic regression for further analysis. In multivariate analysis, marital status, cardiac illness, and asthma symptom control level were significantly associated with depression. The odds of developing depression among single asthma patients were increased by 1.63 at 95% CI [1. 8, 3.493]. Depression among asthma patients who had comorbid cardiac illness was 6.2 times higher than those who do not have at CI [1.145, 24.109]. The prevalence of depression among uncontrolled asthma patients was 8 times higher than those with well-controlled asthma at CI [1.114, 19.025] (Table 2).

## 4. Discussion

Depression is considered to be a common presentation in asthma patients. This study aimed to estimate the burden of depression among asthma patients attending government hospitals in Ethiopia. It was found that the prevalence of depression among asthma patients was 21%. Based on a systematic review data, the magnitude of depression was nearly seven percent in the country in the general population. But the review study did not take patients with a comorbidity into account to estimate the prevalence [16]. Depression was found to be higher (43.4%) in tuberculosis, 15.4% in diabetes, and 38.94% in HIV patients, 11.8% in pregnant women, and 28.5% among geriatric population [21–25]. Further, the prevalence of depression was significantly higher among Parkinson patients 57.4% [26]. In the present study, the odds of depression among asthma patients with cardiovascular comorbidity were six times higher than those without comorbidities. The culmination of these findings suggest depression, as a single entity, can be abundant in asthma patients. Continental data from Nigeria reported that high proportion (67.4%) of patients were diagnosed for depression in asthma patients [14]. Nonetheless, the prevalence of depression was somewhat lower than study done in Nigeria. The possible reason for low epidemiology of depression in our study might be due to the application of different tool. In Nigeria study BDI was used but in this study, PHQ-9 was utilized. In addition, other possible reasons for the discrepancy could be different age category. In Nigeria study participants were between 10 and 49 age ranges but in current study patients below 18 years old were not included. Approximately comparative figure was obtained in 2014 in Korea (16.8%) which applied the same tool [27].

In the present study it was found that single marital status was associated with increased burden of depression. This finding is consistent with other studies. For instance, Scot K et al. 2010 have discovered that being married was associated with reduced risk of first onset of most mental disorders [28]. Another cross-sectional study in Nigeria indicated that being alone increases the probability to be depressed [14]. Consequently, exposure to depression escalated the proportion of individuals who terminated their marriage and being separated or divorced [29].

In this study uncontrolled asthma was highly associated with depression. Depression might lead to the poor control of asthma symptoms which might be in part, due to lack of persistence with medications. In addition, depression could be an independent factor for incident asthma attack [30]. A cross-sectional study in Egyptian showed the prevalence of depression was high among those who had uncontrolled asthma. This indicated that the probability of developing depression could be higher among uncontrolled asthmatic patients. In turn, the coincidence of depression could be a risk factor for uncontrolled asthma [15, 31, 32].

### 4.1. Strength and Limitation of the Study

In general, the present study provided an evidence on the prevalence of depression in asthmatic patients. But it has some limitations. The cross-sectional nature of this study cannot allow for making causal inference. In addition, since the current study is facility-based study, the findings cannot be generalized to people in the community who remain undiagnosed or untreated. Moreover, the study area is limited to the capital city; it did not represent the epidemiology of depression among asthmatic patients in Ethiopia. Further, all factors that can affect the prevalence of depression in asthmatic patients may not be elucidated in the current study.

## 5. Conclusion

In the current study, one-fifth of the study participants experienced depression among asthmatic patients. Uncontrolled asthma, comorbid cardiac illness, and widowed patients were important factors which predict depression among asthmatic patients. Routine screening of depression on those have uncontrolled asthma is required. Further, it is better to assess depression for comorbid cardiac illness patients.

## Figures and Tables

**Table 1:** Sociodemographic characteristics among asthma patients at governmental hospital Addis Ababa, Ethiopia, 2017.

**Sociodemographic variable**	**Frequency n(**%**) =405**

**Age category**	

18-28	6(1.5)
29-39	27(6.7)
40-50	100(24.7)
51-61	163(40.2)
>62	109(26.9)

**Sex**	

Male	132(32.6)
Female	273(67.4)

**Religion**	
Orthodox Christian	292(72.1)
Muslim	89(22)
^*∗*^Other	24(5.9)

**Ethnicity**	
Amhara	135(33.3)
Tigre	61(15.1)
Oromo	180(44.4)
^*∗∗*^Others	29(7.2)

**Marital status**	

Married	282(69.6)

Widowed	82(20.2)

Single	40(6.9)

**Educational status**	

Unable to read and Wright	153(37.8)

Elementary	142(35.1)

High school	51(12.6)

College diploma	34(8.4)

First degree and above	25(6.2)

Occupational status	
Housewife	191(47.2)
Retired	149(36.79)
Self-employee	65(16)

**Table 2:** Bivariate and multivariate logistic analysis result of study subjects among asthma patients at governmental hospitals Addis Ababa, Ethiopia, 2017.

**Variable **	**Depression**		**COR(CI) **	**AOR(CI)**	**P value**
	Yes	No			

**Marital status**					
Married	58(14.32	224(55.31)	1	1	
Widowed	15(3.71)	67(16.54)	1.598(0.769,3.323)	0.082(0.001,4.690)	0.225
**Single **	12(2.96)	28(7.16)	1.865(1.461,2.623)	1.63(1. 8-3.493)	**0.01**

**Cardiac illness**					
No	12(2.96)	46(11.34)	1	1	
**Yes **	**8(1.97)**	**12(2.96)**	**2.556(0.853,7.656)**	**6.249(1.145,24.109)**	**0.03**

**Asthma symptom control level**					
Well controlled	10(2.47)	99(24.44)	1	1	
Partly controlled	28(6.91)	142(35.6)	1.952(0.907,4.200)	1.020(0.163,6.360)	0.983
**Uncontrolled**	**47(11.60)**	**79(19.51) **	**5.890(2.799,8.392)**	**7.884(1.114,19.025)**	**0.04**

Sex					
Male	18(4.44)	114(28.15)	1	1	1
Female	67(16.54)	206(50.86)	2.060(1.167,3.637)	5.570(0.00,6.495)	0.988

Social support					
Strong	12(2.96)	44(10.86)	1	1	
Poor	60(14.81)	151(37.28)	1.457(0.720,2.948)	2.141(0.295,15.548)	0.452
Moderate	13(3.21)	125(30.86)	0.381(0.162,0.898)	0.099(0.006,1.559)	0.100

Substance use					

Yes	2(0.49)	22(5.4)	1	1	

No	83(21.23)	298(73.58)	3.064(0.706,13.296)	0.00(0.00,0.001)	0.998

Prednisolone					
Yes	6(1.48)	44(10.86)	1	1	
No	79(19.51)	276(68.14)	2.099(0.863,5.106)	0.154(0.007,3.233)	0.228
Beclomethasone					

No	57(14.7)	176	1	1	

Yes	28(6.91)	141	0.624(0.377,1.032)	0.611(0.109,3.418)	0.575
Past Psychiatric history					
Yes	2(0.49)	2(0.449)	1	1	
No	83(20.49)	318(78.52)	0.261(0.036,1.881)	0.147(0.002,10.664)	0.380

Statistically significant at p value<0.05; Hosmer and Lemeshow test at p value=0.192.

## Data Availability

All data generated or analyzed during this study are included in this manuscript.

## Acronyms

AOR: Adjusted odds ratio
BDI: Beck Depression Inventory
BSc: Bachelor of Science
CI: Confidence interval
COR: Crude odds ratio
CES-D: Center for Epidemiological Studies Depression Scale
DSM: Diagnostic Statistical Manual
GINA: Global Initiative for National Asthma
MDE: Major Depressive Episode
PHQ: Patient Health Questionnaire
SD: Standard deviation
ST: Saint
UOG: University of Gondar
USA: United State of America
WHO: World Health Organization.
